# Molecular signatures of soil-derived dissolved organic matter constrained by mineral weathering

**DOI:** 10.1016/j.fmre.2022.01.032

**Published:** 2022-03-02

**Authors:** Ying-Hui Wang, Peng Zhang, Chen He, Jian-Chun Yu, Quan Shi, Randy A. Dahlgren, Robert G.M. Spencer, Zhi-Bing Yang, Jun-Jian Wang

**Affiliations:** a State Environmental Protection Key Laboratory of Integrated Surface Water-Groundwater Pollution Control, School of Environmental Science and Engineering, Southern University of Science and Technology, Shenzhen 518055, China; b Guangdong Provincial Key Laboratory of Soil and Groundwater Pollution Control, School of Environmental Science and Engineering, Southern University of Science and Technology, Shenzhen 518055, China; c State Key Laboratory of Water Resources and Hydropower Engineering Science, Wuhan University, Wuhan 430072, China; d State Key Laboratory of Heavy Oil Processing, China University of Petroleum, Beijing 102249, China; e Shanghai Engineering Research Center of Hadal Science and Technology, College of Marine Sciences, Shanghai Ocean University, Shanghai 201306, China; f Department of Land, Air and Water Resources, University of California Davis, Davis 95616, United States; g Department of Earth, Ocean and Atmospheric Science, Florida State University, Tallahassee 32306, United States

**Keywords:** Soil organic matter, Dissolved organic matter, Chemical composition, Molecular signature, Mineral weathering

## Abstract

Dissolved organic matter (DOM) in soils drives biogeochemical cycling and soil functions in different directions depending on its molecular signature. Notably, there is a distinct paucity of information concerning how the molecular signatures of soil DOM vary with different degrees of weathering across wide geographic scales. Herein, we resolved the DOM molecular signatures from 22 diverse Chinese reference soils and linked them with soil organic matter and weathering-related mineralogical properties. The mixed-effects models revealed that the yields of DOM were determined by soil organic carbon content, whereas the molecular signature of DOM was primarily constrained by the weathering-related dimension. The soil weathering index showed a positive effect on the lability and a negative effect on the aromaticity of DOM. Specifically, DOM in highly weathered acidic soils featured more amino sugars, carbohydrates, and aliphatics, as well as less O-rich polyphenols and condensed aromatics, thereby conferring a higher DOM biolability and lower DOM aromaticity. This study highlights the dominance of the weathering-related dimension in constraining the molecular signatures and potential functions of DOM in soils across a wide geographic scale.

## Graphical abstract

**Figure:**
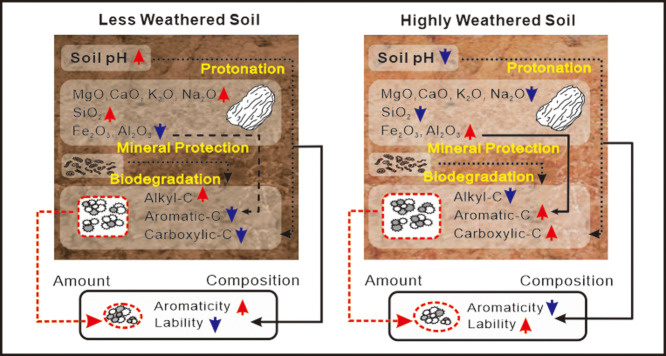

## 1 Introduction

Dissolved organic matter (DOM) in soil consists of a diverse mixture of water-soluble soil organic matter (SOM) molecules that are highly mobile and reactive [1,2]. These molecules play various roles with different capacities in biogeochemical processes involving carbon, nutrients and pollutants [3], [4], [5], [6]. For example, polysaccharide-induced priming may stimulate microbial activity [7,8], whereas tannins may suppress the microbial decomposition process [9,10]. Theoretically, persistence of DOM molecules in soils is controlled by interactions between (i) DOM leaching and desorption that release DOM from plant residues and SOM and (ii) sorption and decomposition that remove DOM [2]. However, there is still no consensus on the dominant factor(s) or dimension(s) driving the yield and molecular signatures of DOM in soil environments.

Recent studies revealed that the microbe-mediated transformation and assembling processes constrained the vertical variations in DOM signatures within soil profiles [11,12] and attributed soil DOM with more recalcitrant signature [13], thereby maintaining relatively persistent DOM signatures in soils [11]. However, molecular variations of soil DOM from distinct geographical regions are primarily attributed to variations in geographical climate conditions and soil clay content [14]. Also, both geographical and edaphic factors can drive soil microbial diversity [15]. Soil weathering condition is highly related to geographical climate conditions, such as temperature and precipitation, and could be a reflection of the soil mineral characteristics [16,17]. Therefore, soil weathering condition could be a promising factor that might simplify the investigation of the mobilization process of DOM in soils. To date, it remains uncertain whether and how mineralogical properties (e.g., mineral elemental composition and weathering indicators) and SOM (e.g., SOM content and composition) may interact to constrain DOM yield and signatures across a diversity of soils. Although optical parameters of DOM based on absorbance and fluorescence spectroscopy have recently been linked to DOM molecular formulae in aquatic environments [18], [19], [20], their linkages to DOM molecular signatures in soil environments have not been rigorously investigated. Thus, improving our understanding of the molecular and optical signatures of DOM from diverse soils across a wide geographic scale, and their constraints, are important goals for unifying conceptual models of DOM dynamics and functions across global terrestrial ecosystems.

Herein, we studied the molecular and optical properties of soil-derived DOM from a set of reference soils with diverse climatic/weathering gradients across China. The reference soils were specifically selected, instead of randomly collected, to represent a range of soil characteristics representative of the large geographical region of China. As the reference soils are commercially available, they allow future studies of DOM signatures and functions on a consistent set of soils. Those reference soils were classified into six soil taxonomic orders based on USDA Soil Taxonomy and represent a soil developmental/weathering sequence: Entisols < Inceptisols ≈ Aridisols < Mollisols < Alfisols < Ultisols [21]. The detailed mineral elemental composition combined with the SOM composition provided a powerful approach to quantitatively examine how weathering-defined soil mineralogy and SOM properties affect the molecular signatures of soil-derived DOM and assess the molecular-level interpretation of the commonly used optical parameters for soil-derived DOM. Here, we aim to identify the hitherto unknown association between specific molecular classes and soil-derived DOM fluorescence, and to distinguish the different regulating effects of SOM and mineral elemental properties on DOM quantity and quality in soils across a large weathering gradient.

## 2 Methods

### 2.1 Soil preparation and characterization

We used 22 Chinese national standard soils (GBW07401−GBW07385) purchased from https://www.gbw-china.com. These soil samples were collected from 15 different provinces (Fig. S1) and had been homogenized by gridding for commercial supplies. Standard soil characterization metrics and analytical methodologies are provided in the standard soil certificates, and the selected metrics used in this paper are reported in Table S1. The selected standard soils had wide ranges of pH (5.4−9.4), soil organic carbon (SOC) content (0.3−1.8%), mineral elemental composition (e.g., SiO_2_, 32.7−74.7%; Al_2_O_3_, 10.3−29.3%; Fe_2_O_3_, 2.0−18.8%;), and weathering degree (Si/Al ratio [22]: 1.9−12.1; chemical index of alteration (CIA) [23]: 53.5−98.4) (see Table S1 for details).

Soil organic carbon composition from different functional groups was assessed by solid-state ^13^C nuclear magnetic resonance (^13^C NMR) spectroscopy. Briefly, 6 g soil was repeatedly washed with 7 treatments of 10% hydrofluoric acid. The residues were washed to a neutral pH and analyzed on a Bruker AVANCE III 600 spectrometer at a resonance frequency of 150.9 MHz. The 4 mm magic angle spinning probe packed with powdered samples spun at a rate of 12 kHz. The contact time was 4 ms, and the recycle delay was set at 2 s for measurement [24]. ^13^C NMR spectra were divided into 7 regions: 0–45 ppm for alkyl C; 45–60 ppm for *N*-alkyl C and methoxyl groups; 60–95 ppm for *O*-alkyl C; 95–110 ppm for di-*O*-alkyl C; 110–145 ppm for aromatic C; 145–165 ppm for *O*-aromatic C; and 165–210 ppm for carboxyl and carbonyl groups [25], [26], [27]. Alkyl/*O*-alkyl C ratio and area ratio of 70–75 to 52–57 ppm was calculated to indicate the decomposition degree and bioavailability of soil organic matter [28].

### 2.2 DOM preparation and optical characterization

Soil DOM was extracted by shaking 10 g soil in 200 mL Milli-Q water at 200 rpm and 20 °C for 2 h. The suspension was filtered through 0.45 μm polyether sulfone membrane (Millipore) and stored at 4 °C prior to further analyses.

General water quality and optical characteristics were analyzed based on our previous report [29]. Specifically, pH was analyzed with a benchtop meter HQ440d and Intellical™ PHC201 pH probe. Dissolved organic carbon (DOC) and total dissolved nitrogen (TDN) concentrations were measured with a TOC-L CSH/CSN (Shimadzu, Japan), and the ratio of DOC to TDN (DOC/TDN) was calculated. Absorbance and three-dimension excitation-emission matrix (3D-EEM) fluorescence spectra were acquired by an Aqualog® spectrophotometer (Horiba, Japan).

We calculated the carbon-specific UV-absorbance at 254 nm (SUVA_254_) and the ratio of absorption at 250 and 365 nm (E2/E3 ratio) to indicate the DOM aromaticity and the apparent molecular weight, respectively [30,31]. Parallel factor (PARAFAC) analysis was used to decompose the fluorescence signal [32]. Two “humic-like” (C1 and C2) and one protein-like (C3) components explained 99.6% of the variance and were validated using split-half analysis with four splits. Fluorescence-based indices, including fluorescence index (FI), humification index (HIX), and biological index (BIX), were calculated to assess the DOM sources, humification degree, and the contribution of autochthonous DOM [[33], [34], [35]].

### 2.3 FT-ICR MS analysis

Soil DOM was solid-phase extracted (SPE) on a Bond Elut PPL (100 mg PPL in 3mL cartridge; Agilent, USA) as described in Wang et al [36]. SPE extracts were analyzed on a 9.4 Tesla FT-ICR MS (Bruker, Germany) using standard methods described in He et al [37]. Specifically, the SPE DOM extracts with DOC concentration at about 50 mg/L were injected into ESI sources at a rate of 250 μL/h. The typical operating conditions for negative-ion ESI analysis were: 3.5 kV spray shield voltage, 4.0 kV capillary column introduced voltage, -320 V capillary column end voltage, and ions accumulated in the collision cell for 0.2 s then transferred into the ICR cell with a 1.2 ms time-of-flight. The ion transformation parameter for the quadrupole was optimized at *m/z* 300. The mass range was *m/z* 200–800. A total of 128 scans with 2 M word size were accumulated to enhance the signal-to-noise ratio. The spectra were calibrated with an internal calibration list that provided a mass accuracy of 0.2 ppm or higher throughout the mass range of interest. Peaks with a signal-to-noise ratio greater than 6 were assigned using in-house software. The mass accuracy window was set to 1.0 ppm. The elemental combination for each formula was set as: ^12^C_1–60_, ^1^H_1–120_, ^14^N_0–3_, ^16^O_0–30_, and ^32^S_0–1_. In addition, the elemental formulae should meet the following criteria: (1) the number of H atoms must be at least 1/3 the number of C atoms and cannot exceed 2C + N + 2; (2) the sum of H and N atoms must be even (the nitrogen rule); and (3) the number of N or O atoms cannot exceed the number of C atoms. Formulae assigned with mass accuracy <1.0 ppm were categorized into four elemental groups (CHO, CHON, CHOS, and CHONS) and five biochemical groups into different groups based on elemental composition: lipid (O/C ≤ 0.6; H/C ≥ 1.32; N/C ≤ 0.126), protein (0.12 < O/C ≤ 0.6; 0.9 < H/C < 2.5; 0.126 ≤ N/C ≤ 0.7 or 0.6 < O/C ≤ 1.0; 1.2 < H/C < 2.5; 0.2 < N/C ≤ 0.7), amino sugar (O/C ≥ 0.61; H/C ≥ 1.45; 0.07 < N/C ≤ 0.2), carbohydrate (O/C ≥ 0.8; 1.65 ≤ H/C < 2.7; *N* = 0), and phytochemical (O/C ≤ 1.15; H/C < 1.32; N/C < 0.126) [38]. Modified aromaticity index (AI_mod_) was calculated to assess the degrees of aromaticity [39], and nominal oxidation state of carbon (NOSC) was calculated to indicate the relative nominal oxidation state of organic matter [18]. The percentage of labile compounds above the molecular lability boundary (%MLB_L_, H/C≥1.5) was calculated to estimate the abundance of labile organic matter [40].

### 2.4 Data management and statistical analyses

We applied non-metric multidimensional scaling (NMDS) based on Bray-Curtis distance for permute fitting of molecular variables [15,18]. Optical indices were fit to the ordination to reveal molecular characteristics that covaried with those properties. Significance was assessed through a Monte Carlo test (999 permutations), and variables with a significance level of *P*<0.1 were shown. NMDS was calculated using the vegan package in R software. Spearman correlation was calculated between the sum-normalized intensity of peaks that were present in at least 90% of the samples and optical indices, SOM composition and elemental constituents [4,14,20]. Formulae with significant correlations (*P*<0.1) to other indexes were shown in van Krevelen plots. Linear correlations between multiple proxies were assessed using SPSS. Structural equation models (SEM) evaluated complex network relationships among soil properties and the mobilization of soil DOM. Before SEM analysis, we conducted principal components analysis based on ^13^C NMR results to generate a multivariate index with the loading on the first principal component axis representing the SOM composition. The first principal component associated with high alkyl C abundance and the low area ratio of 70–75/52–57 ppm explained 77% of the total variance. SEM analyses were performed by AMOS 21.0, and model fit was evaluated by model χ^2^ test and the root-mean-square error of approximation. A database of measured parameters is available from the corresponding author upon request. All other data are included in Supplementary Information files.

## 3 Results and discussion

### 3.1 DOC yield from soils driven by SOC content

DOC yield ranged from 0.1 to 1.3 mg-C/g-soil and was linearly correlated with SOC content (*r* = 0.87, *P*<0.01), but not with the relative abundances of SOC fractions or soil elemental composition (*P*>0.05; Fig. S2). Based on the linear relationship, every 1% increase in SOC content resulted in an increase of approximately 0.68 ± 0.08 mg DOC yield from 1 g soil. Although DOM polarity (e.g., abundance of carboxyl C in SOC) and pH should theoretically affect the solubility of SOC [2], DOC yield from soils was not correlated with either of these factors, but rather appeared to be strongly controlled by SOC content across our wide range of soil types.

### 3.2 Linkages between molecular and optical characters of soil-derived DOM

**Fig. 1:**
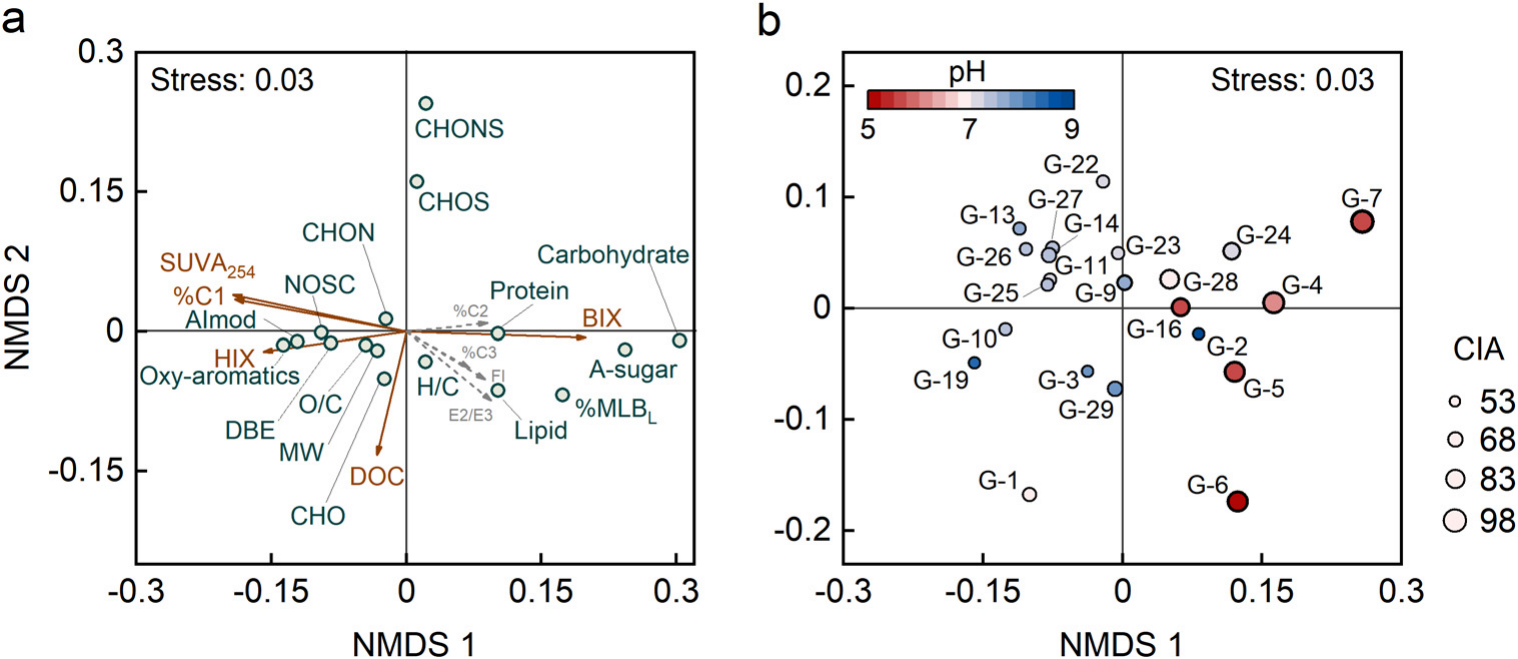
**Multivariate analysis of molecular and optical signatures of DOM using non-metric multidimensional scaling.** (a) Ordination was based on Bray-Curtis dissimilarities of variables derived from FT-ICR MS molecular composition (*k*=3, stress=0.03). Optical indices were fit to the same ordination over 999 permutations. Variables (% C2, %C3, and FI) with significance levels >0.1 are shown in light gray, and others with *P*<0.1 are shown in brown. (b) Samples are color-coded based on the pH gradient, and sized by the weathering degree indicated by the chemical index of alteration (CIA). Abbreviations: SUVA_254_, specific ultraviolet absorbance at 254 nm; HIX, humification index; E2/E3, absorbance at 254 nm divided by the absorbance at 365 nm; BIX, biological index; FI, fluorescence index; %C1–%C3, relative abundances of fluorescence components C1–C3.

FT-ICR MS analysis resolved 9418 molecular formulae across all samples. We used NMDS to explore linkages between molecular characteristics of DOM and widely used optical indices, such as SUVA_245_, E2/E3 ratio, FI, HIX, BIX and the fractions of fluorescent DOM components (%C1, %C2 and %C3; details in Fig. S3). Results showed that DOM from the less weathered alkaline soils (pH = 7.8 ± 0.4; CIA =59.6 ± 3.8; Si/Al = 8.6 ± 1.1) distributed at the negative end of the first dimension and had high SUVA_254_, HIX and %C1, as well as high aromaticity (AI_mod_) and high portion of condensed aromatic and polyphenolic compounds (Fig. 1). These findings indicate that the most abundant “terrestrial humic-like” fluorescent DOM (C1) in soils is highly aromatic, oxygen-rich and has a relatively high molecular weight, similar to findings from natural waters, such as boreal lakes [18], rivers [19] and seawater [20]. Conversely, DOM from the highly weathered acidic soils (pH = 6.0 ± 0.4; CIA =91.2 ± 5.9; Si/Al = 4.1 ± 1.5) was found at the positive end of the first dimension and had high %MLB_L_, as well as the high portion of peptide-like and aliphatic components. Those characteristics are consistent with high BIX that suggests a high relative contribution of microbial-derived DOM [4,40]. Therefore, DOM from the highly weathered acidic soils is more likely be biolabile.

**Fig. 2:**
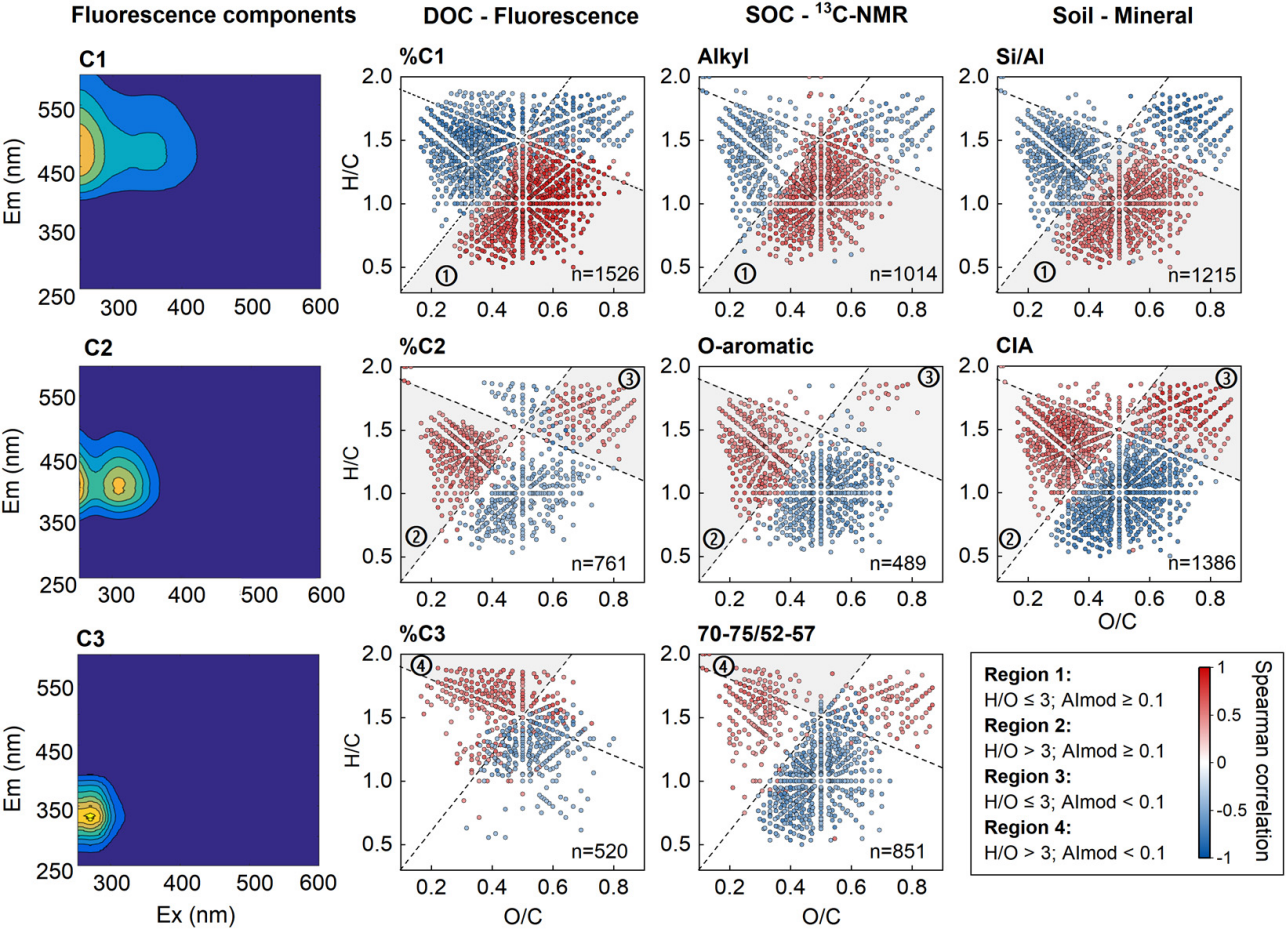
**Molecular associations between individual molecules and three fluorescence components, soil organic matter composition and soil mineral characteristics.** FT-ICR MS compounds with significant Spearman correlations (*P*<0.1) with other indexes are plotted in van-Krevelen diagrams. Red color indicates a positive correlation, and blue color indicates a negative correlation. Regional lines are combination of AI_mod_ = 0.1 and H/O = 3.

To better resolve the possible relationships of DOM molecules to the various fluorescent components, we investigated the relative abundances of fluorescent components with the normalized mass spectral peak intensities of 2087 formulae that occurred in 90% of soil samples (accounting for 64–75% of the total intensity for each sample). Based on the projection of Spearman correlation coefficients on van Krevelen plots (Fig. 2), the distribution of associated formulae in soils did not suggest a direct connection with the elemental or biochemical groups. Instead, the fluorescent-component-related formulae clustered in four regions: (i) “terrestrial-humic-like” C1 related formulae in Region I (H/O ≤3 and AI_mod_ ≥0.1), which contains large amounts of O-rich highly unsaturated compounds, polyphenols and condensed aromatics; (ii) “microbial-humic-like” C2 related formulae in Region II (H/O >3 and AI_mod_ ≥0.1), which contains large amounts of O-poor highly unsaturated aliphatics and aromatics; (iii) “microbial-humic-like” C2 related formulae in Region III (H/O ≤3 and AI_mod_ <0.1), which contains large amounts of amino sugars and carbohydrates; and (iv) “protein-like” C3 related formulae in Region IV (H/O >3 and AI_mod_ <0.1), which contains large amounts of saturated aliphatic and peptide-like formulae. After dividing the 2087 formulae into these four regions, we calculated the arithmetic mean numbers of the C, H, O, N, and S for each region, and the average formulae belonging to Regions I–IV were C_16.71_H_17.13_O_8.12_N_0.77_S_0.00_, C_19.29_H_25.73_O_6.18_N_0.34_S_0.00_, C_14.48_H_22.04_O_9.33_N_1.01_S_0.01_, and C_16.19_H_27.18_O_6.66_N_0.80_S_0.03_, respectively (Table S2). The relative abundances of the common formulae in the four regions strongly covaried with the relative abundances of total formulae clustered in the corresponding regions (Fig. S4). These four clusters showed distinct patterns contrary to fluorescent-component-related formulae clusters identified in lakes [18]. Importantly, this analysis highlights that similar fluorescent signals may have completely distinct molecular identities in DOM from terrestrial and aquatic environments.

### 3.3 Linking DOM signatures to SOM and mineral elemental constraints

**Fig. 3:**
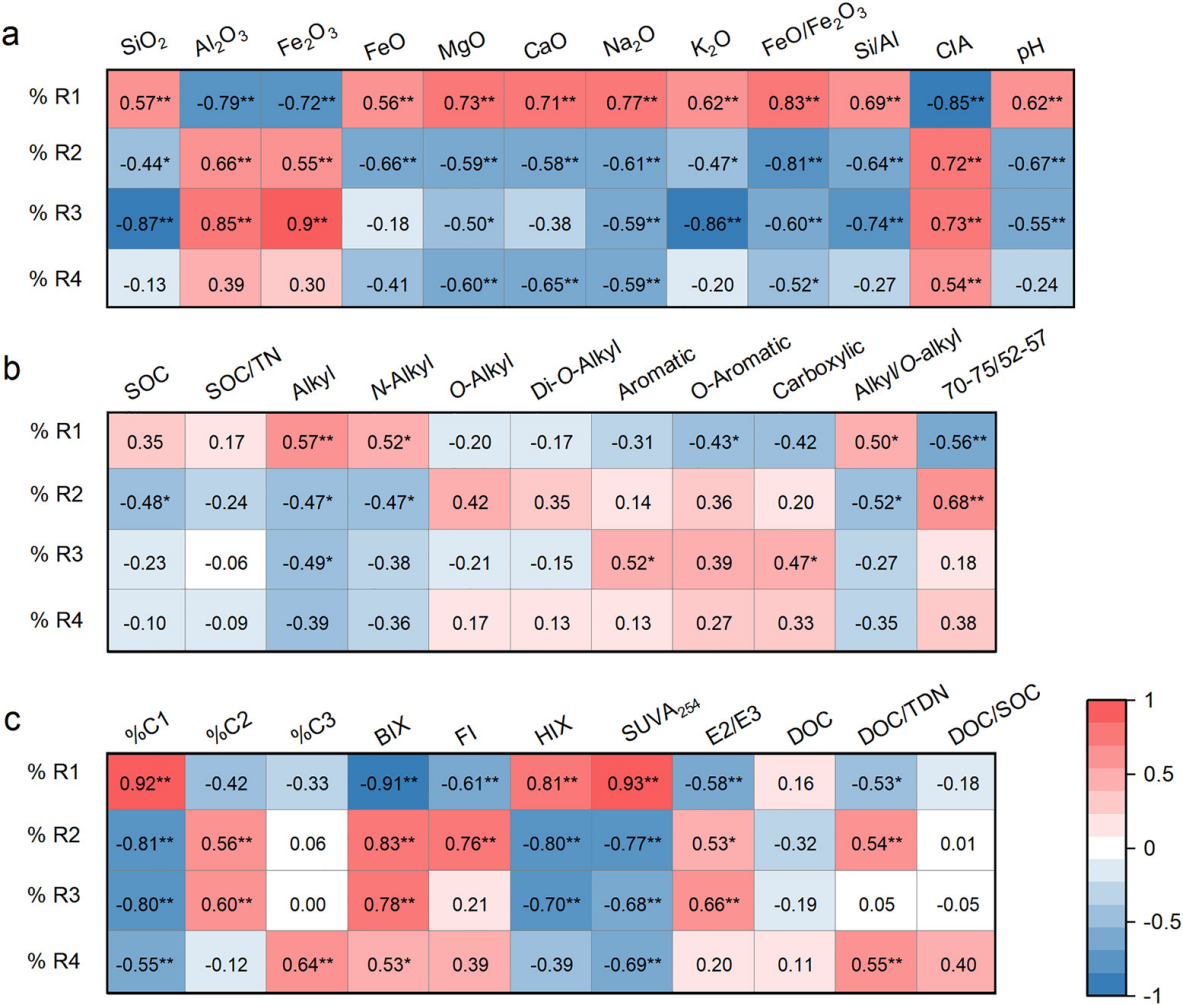
**Heat maps showing the relationship between the relative abundance of all soil dissolved organic matter formulae clustered in four regions (see region definition in**Fig. 2**legend) and soil parameters.** Corrleations with categorical variables of (a) soil mineral properties, (b) soil organic carbon characters, and (c) soil dissolved organic matter characters. Correlations are scaled from red for a positive correlation to blue for a negative correlation. Significance levels: * *P*<0.05, ** *P*<0.01.

To explore potential SOM and mineral elemental constraints on soil DOM characteristics, we applied Spearman correlation analyses between individual formulae and soil properties (examples in Fig. 2 and full analysis in Figs. S5–S7) and linear correlation analyses (Fig. 3) of the relative abundances of DOM formulae in the four regions (%R1−%R4) versus soil/mineralogical properties.

The “terrestrial-humic-like” C1-related Region I contains formulae clusters that positively correlated with SOC content, pH, alkyl C, N-alkyl C, alkyl/*O*-alkyl ratio, SiO_2_, MgO, CaO, Na_2_O, and FeO contents, and FeO/Fe_2_O_3_ and Si/Al ratios, and negatively correlated with *O*-aromatic C, carboxyl C, an area ratio of 70–75/52–57 ppm, Al_2_O_3_ and Fe_2_O_3_ contents and CIA (Figs. S5–S7). The relative abundance of formulae in Region I (%R1) showed similar correlations with these indices (Fig. 3). Therefore, the more highly weathered soils having a higher CIA and lower Si/Al ratio and pH values were found to contain less O-rich DOM molecular formulae in Region I. This phenomenon can be explained by ligand-exchange complexation of DOM molecules with high aromaticity and acidic functional groups at surfaces of Al/Fe (hydr)oxides prevalent in highly weathered and acidic soils [41], [42], [43]. This strong complexation can reduce the possible dissolution of the terrestrial humic-like DOC components and lead to the long-term accumulation of O-aromatic C in the SOC. Additionally, in the strongly weathered soils with relatively low pH, the highly oxygenated DOM molecular formulae in Region I tend to protonate and cause lower solubility and DOM yields [44,45].

Contrasting with Region I, the “microbial-humic-like” C2-related Regions II and III contained formulae clusters that showed negative correlations with the degree of mineral weathering (e.g., pH, elemental composition, Si/Al ratio and CIA; Figs. S5–S7). Relative abundances of DOM molecular formulae in Regions II and III (%R2 and %R3) also showed significant correlations with these parameters (Fig. 3). Clearly, the molecular formulae in these two regions were also mainly constrained by the weathering-related dimension. In highly weathered soils, these constituents tend to be weakly sorbed by Al/Fe (hydr)oxides [41], [42], [43]. Thus, these O-poor highly unsaturated compounds and aromatics (Region II) and the amino sugars and carbohydrates (Region III) account for higher proportions of the DOM pool. The O-poor molecular formulae in Region II that were significantly correlated with BIX, FI and DOC/TDN may have higher contributions from freshly produced microbial byproducts [46]. The molecular formulae in Region III contained a significant amount of amino sugars and carbohydrates that should not contribute to the fluorescent signals of the “microbial-humic-like” C2 fraction due to the lack of a conjugation structure [47]. Nevertheless, amino sugars are specifically sourced from microbes and their high correlation with “microbial-humic-like” C2 support the feasibility of using this fluorescence signal to indicate microbial contributions to SOC.

The “protein-like” C3 related Region 4 contained substantial amounts of saturated aliphatic and peptide-like molecular formulae, which supports the possibility of using this fluorescence signal as an indicator of protein contributions. Formulae in this region negatively correlated with CaO and MgO contents and SUVA_254_, and positively correlated with BIX and DOC/TDN (Figs. S5–S7). These relationships suggest that they are important contributor to the non-aromatic fraction and are influenced by localized nutrient conditions. Nevertheless, this region contains only a few molecular formulae that correlated with weathering-related parameters, such as Si/Al ratio and CIA, suggesting that this region is less affected by mineral weathering than other biochemical regions.

Overall, the four clusters that linked to the optical parameters of DOM showed distinct tendencies for being released from SOM, interacting with Al/Fe (hydr)oxides, and being microbiologically processed based on their relations with various soil properties. Importantly, the mineral weathering-related dimension (e.g., pH, elemental composition and mineral weathering indices) was identified as the most important constraint on the major molecular signatures of soil-derived DOM, especially those in Regions I–III, but not those in Region IV.

### 3.4 Geochemical, environmental and ecological significance

**Fig. 4:**
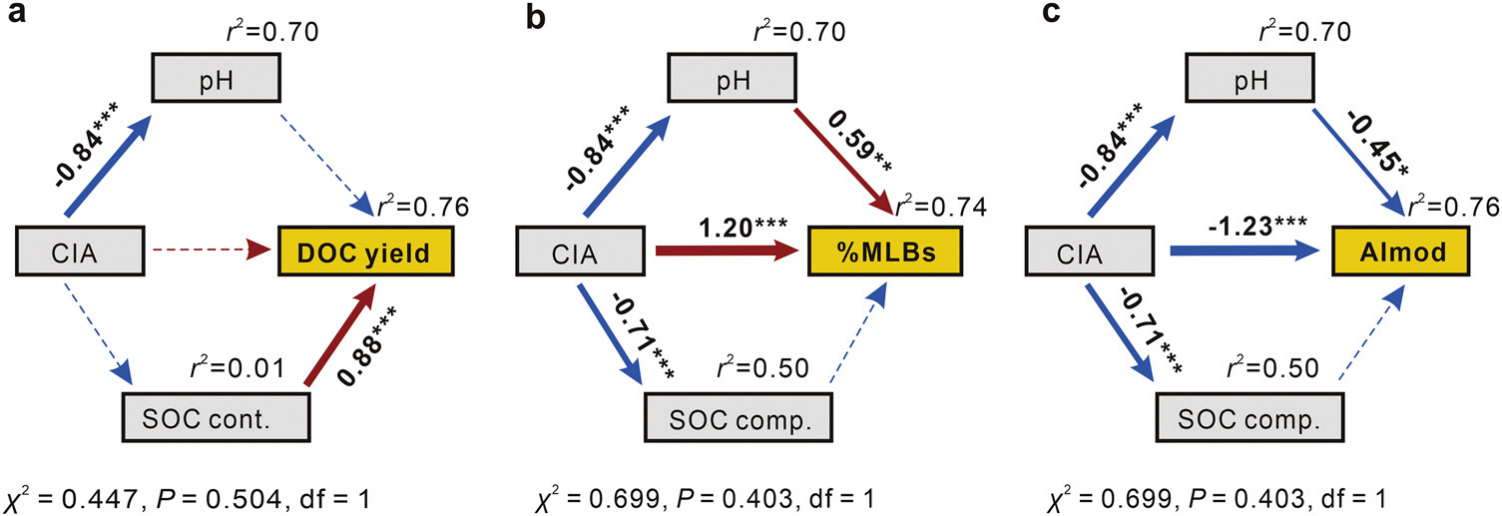
**Structure equation models examining the multivariate effects of soil elemental and soil organic composition on (a) soil DOC yield and (b, c) soil DOM composition.** Arrows indicate the hypothesized path of causation, while dotted arrows indicate insignificant pathways (*** *P*<0.001; ** *P*<0.01; * *P*<0.05). Arrow width is proportional to the strength of the relationship, while red and blue arrows indicate positive and negative relationships, respectively. The numbers adjacent to arrows are standardized path coefficients, which reflect the effect size of the relationship. SOM is represented by the first component from the principal component analysis based on ^13^C NMR.

Quantity and quality of soil DOM (e.g., bioavailability and aromaticity) are critical factors regulating several soil functions, such as fueling soil microbes for carbon/nutrient cycling with labile substrates and transporting pollutants via cation-π and π-π interactions [3,4,48]. We used structural equation models (SEM) based on the relationships between DOC with SOC and soil elemental characteristics (Figs. S2, S8, and S9) to assess the critical factors controlling DOC yield, %MLB_L_ and AI_mod_. The models explained a high degree of variance in the quantity and quality of soil DOM (*r*^2^>0.7) across the diversity of soils. The models confirmed the important role of SOC content as the dominant driver of DOC yield (Fig. 4a). While mineral weathering (indicated by CIA) can affect pH and SOC composition, mineral weathering rather than pH or SOC composition had the strongest direct effect on both %MLB_L_ and AI_mod_ of DOM (Fig. 4b and c). Accordingly, the soils with a greater degree of weathering yielded more biolabile DOM components with lower DOM aromaticity.

**Fig. 5:**
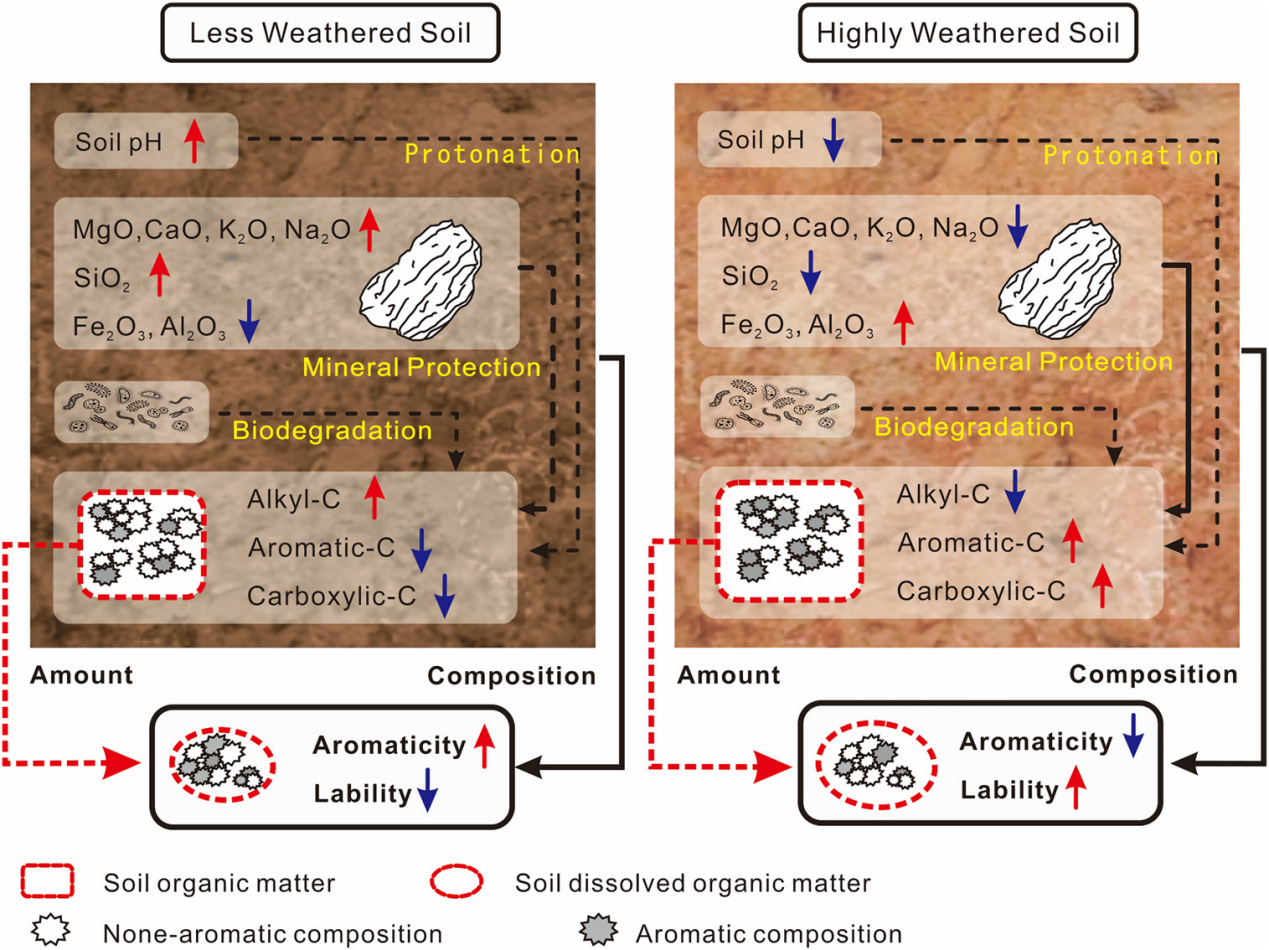
**Conceptual model showing the impacts of soil weathering and soil organic matter on the quantity and quality of soil dissolved organic matter.** The characters of less weathered and highly weathered soils were extracted based on redundancy analysis and principal component analysis (Figs. S10 and S11). Positive and negative responses are indicated by red and blue arrows, respectively. With increasing weathering degree, the loss of base cations (Mg^2+^, Ca^2+^, Na^+^, and K^+^) increased, and silica (Si) preferentially dissolves/leaches versus Al and Fe, leaving Al/Fe (hydr)oxides to adsorb and preserve aromatic carbon. In addition, a lower pH caused by intense weathering/leaching may increase the protonation of phenolic DOM and decreased the solubility of soil aromatic carbon. With strong processing by microbes, DOM from less weathered soil is featured with less aromaticity and a higher amount of microbial processed components, and therefore has a lower molecular weight and higher lability. Conversely, the DOM concentration is primarily determined by the amount of soil organic matter.

Our study highlights the dominant role of SOM content and soil elemental/mineralogical characteristics in regulating the yield and molecular signatures of soil-derived DOM, respectively (Fig. 5). Within highly weathered soils, aromatic carbon constituents tend to be adsorbed and chemically protected by sorption to Al/Fe (hydr)oxides, thereby accumulating in soils. Additionally, a lower aromatic content of soil DOM can make it more available to microbes and thus more biodegradable. The molecular signatures of soil-derived DOM and their variations with soil properties provide a fundamental dataset for understanding, modeling and optimizing specific DOM functions across various geographical areas using soil-mapping inventories. While the present findings are derived from a limited series of reference soils (22 diverse soils), this study provides a conceptual framework for future investigations to expand the geographical extent of soil characteristics to further unify the interpretation and modeling of molecular signatures and functions of soil-derived DOM.

## Data availability

The data supporting the findings of this study is available within supplementary information. The detailed molecular data is archived in open research data repository under Science Data Bank (http://www.doi.org/10.11922/sciencedb.01064).

## CRediT authorship contribution statement

**Ying-Hui Wang:** Data curation, Formal analysis, Writing – original draft, Writing – review & editing. **Peng Zhang:** Data curation, Formal analysis. **Chen He:** Data curation, Formal analysis. **Jian-Chun Yu:** Data curation, Formal analysis. **Quan Shi:** Data curation, Formal analysis. **Randy A. Dahlgren:** Data curation, Writing – original draft, Writing – review & editing. **Robert G.M. Spencer:** Data curation, Writing – original draft, Writing – review & editing. **Zhi-Bing Yang:** Data curation, Writing – original draft, Writing – review & editing. **Jun-Jian Wang:** Conceptualization, Investigation, Funding acquisition, Data curation, Writing – original draft, Writing – review & editing.

## Declaration of competing interest

The authors declare that they have no conflicts of interest in this work.

## Appendix Supplementary materials

Image, application 1
