# Supplementary material for: Molecular signatures of soil-derived dissolved organic matter constrained by mineral weathering
Source: Fundam Res. 2022 Mar 2;3(3):377–83. doi: 10.1016/j.fmre.2022.01.032 (PMC11197591; doi:10.1016/j.fmre.2022.01.032)
Supplement: Supplementary file 1 [file mmc1.docx]

**Supplementary Information**

**Molecular signatures of soil-derived dissolved organic matter constrained by mineral weathering**

**Authors:** Ying-Hui Wang ^a,b,c^, Peng Zhang ^a,b^, Chen He ^d^, Jian-Chun Yu ^e^, Quan Shi ^d^, Randy A. Dahlgren ^f^, Robert G. M. Spencer ^g^, Zhi-Bing Yang ^c^, Jun-Jian Wang ^a,b,^*

**Affiliations:**

^a^ *State Environmental Protection Key Laboratory of Integrated Surface Water-Groundwater Pollution Control, School of Environmental Science and Engineering, Southern University of Science and Technology, Shenzhen 518055, China*

^b^ *Guangdong Provincial Key Laboratory of Soil and Groundwater Pollution Control, School of Environmental Science and Engineering, Southern University of Science and Technology, Shenzhen 518055, China*

^c^ *State Key Laboratory of Water Resources and Hydropower Engineering Science, Wuhan University, Wuhan 430072, China*

^d^ *State Key Laboratory of Heavy Oil Processing, China University of Petroleum, Beijing 102249, China*

^e^ *Shanghai Engineering Research Center of Hadal Science and Technology, College of Marine Sciences, Shanghai Ocean University, Shanghai 201306, China.*

^f^ *Department of Land, Air and Water Resources, University of California Davis, Davis， 95616, United States*

^g^ *Department of Earth, Ocean and Atmospheric Science, Florida State University, Tallahassee, 32306, United States*

* Contact of the Corresponding Author, Email: wangjj@sustech.edu.cn

**The supplementary information contains:**

1. **Supplementary tables:**

Table S1: Physiochemical properties of soil samples.

Table S2: Regional signatures of 2087 formulae occurring in 90% of soil samples.

1. **Supplementary figures**

Figures S1：Map showing the locations of the 22 Chinese reference soils.

Figures S2: Relationships of the dissolved organic carbon yield with soil organic carbon content, ^13^C-NMR compositions, soil elemental composition and soil pH.

Figure S3: Fluorescence signatures of three PARAFAC components.

Figure S4: Correlations for the relative abundances of the common DOM formulae versus all soil DOM formulae in four characteristic regions.

Figure S5: Molecular associations between individual molecules and soil elemental composition.

Figure S6: Molecular associations between individual molecules and soil DOM properties.

Figure S7: Molecular associations between individual molecules and soil organic matter properties.

Figure S8: Relationships between the relative abundance of labile molecules above the molecular lability boundary (% MLB_L_) with soil organic carbon content, ^13^C-NMR compositions, soil elemental composition and soil pH.

Figure S9: Relationships between the modified aromatic index (AI_mod_) with soil organic carbon content, ^13^C-NMR compositions, soil elemental composition and soil pH.

Figure S10: Redundancy analysis showing the relationship between soil elemental composition, SOM composition and soil DOM optical characters.

Figure S11: Principle component analyses of soil elemental composition, soil organic matter components and soil DOM characters.

Table S1: Physiochemical properties of soil samples.

|  |  | **GSS1** | **GSS2** | **GSS3** | **GSS4** | **GSS5** | **GSS6** | **GSS7** |
| --- | --- | --- | --- | --- | --- | --- | --- | --- |
| **Elemental**  **Content**  **(%)** | SiO_2_ | 62.60 | 73.35 | 74.72 | 50.95 | 52.57 | 56.93 | 32.69 |
|  | Al_2_O_3_ | 14.18 | 10.31 | 12.24 | 23.45 | 21.85 | 21.23 | 29.26 |
|  | Fe_2_O_3_ | 5.19 | 3.52 | 2.00 | 10.3 | 12.62 | 8.09 | 18.76 |
|  | MgO | 1.81 | 1.04 | 0.58 | 0.49 | 0.61 | 0.34 | 0.26 |
|  | CaO | 1.72 | 2.36 | 1.27 | 0.26 | 0.10 | 0.22 | 0.16 |
|  | Na_2_O | 1.66 | 1.62 | 2.71 | 0.11 | 0.12 | 0.19 | 0.08 |
|  | K_2_O | 2.59 | 2.54 | 3.04 | 1.03 | 1.50 | 1.70 | 0.20 |
|  | FeO | 1.27 | 0.57 | 0.5 | 0.41 | 0.22 | 0.57 | 1.05 |
|  | Si/Al | 7.49 | 12.07 | 10.36 | 3.69 | 4.08 | 4.55 | 1.90 |
|  | CIA | 63.18 | 56.06 | 54.89 | 94.08 | 91.60 | 89.60 | 98.39 |
|  | pH | 7.23 | 9.45 | 8.37 | 6.62 | 6.04 | 5.43 | 6.00 |
| **SOC content**  **(%)** | SOC | 1.80 | 0.49 | 0.51 | 0.62 | 0.32 | 0.81 | 0.64 |
| **SOC**  **composition**  **(R.A.)** | Alkyl | 0.24 | 0.30 | 0.23 | 0.20 | 0.26 | 0.19 | 0.19 |
|  | *N*-Alkyl | 0.09 | 0.13 | 0.11 | 0.09 | 0.09 | 0.08 | 0.09 |
|  | *O*-Alkyl | 0.21 | 0.19 | 0.23 | 0.22 | 0.28 | 0.25 | 0.17 |
|  | Di-*O*-Alkyl | 0.05 | 0.03 | 0.06 | 0.05 | 0.07 | 0.06 | 0.03 |
|  | Aromatic | 0.18 | 0.19 | 0.16 | 0.20 | 0.12 | 0.19 | 0.25 |
|  | O-Aromatic | 0.09 | 0.06 | 0.06 | 0.07 | 0.06 | 0.07 | 0.08 |
|  | Carboxylic | 0.14 | 0.10 | 0.15 | 0.18 | 0.12 | 0.16 | 0.20 |
|  | Alky/O-alkyl | 1.14 | 1.64 | 0.97 | 0.89 | 0.92 | 0.75 | 1.13 |
|  | 70-75/52-57 | 1.57 | 0.93 | 1.36 | 1.75 | 2.15 | 2.55 | 1.38 |
| **DOC content (mg C/g)** | DOC | 1.31 | 0.27 | 0.45 | 0.34 | 0.14 | 0.52 | 0.29 |
| **Absorbance** | SUVA_254_  (L mgC^-1^ m^-1^) | 1.79 | 1.34 | 1.82 | 0.52 | 0.49 | 0.41 | 0.24 |
|  | E2/E3 | 5.04 | 3.64 | 4.28 | 4.35 | 6.85 | 8.45 | 8.89 |
| **Fluorescence** | C1 (R.A.) | 0.42 | 0.32 | 0.40 | 0.19 | 0.17 | 0.20 | 0.12 |
|  | C2 (R.A.) | 0.27 | 0.13 | 0.16 | 0.44 | 0.39 | 0.49 | 0.52 |
|  | C3 (R.A.) | 0.31 | 0.55 | 0.44 | 0.37 | 0.44 | 0.31 | 0.36 |
|  | BIX | 0.47 | 0.67 | 0.54 | 0.84 | 0.79 | 0.88 | 0.91 |
|  | FI | 1.26 | 1.40 | 1.25 | 1.44 | 1.57 | 1.50 | 1.33 |
|  | HIX | 0.85 | 0.73 | 0.80 | 0.43 | 0.37 | 0.48 | 0.38 |

Table S1 continued.

|  |  | **GSS9** | **GSS10** | **GSS11** | **GSS13** | **GSS14** | **GSS16** | **GSS19** |
| --- | --- | --- | --- | --- | --- | --- | --- | --- |
| **Elemental**  **Content**  **(%)** | SiO_2_ | 61.69 | 65.50 | 69.42 | 64.88 | 64.51 | 63.81 | 66.95 |
|  | Al_2_O_3_ | 13.28 | 13.80 | 13.14 | 11.76 | 14.43 | 17.85 | 11.02 |
|  | Fe_2_O_3_ | 4.80 | 4.17 | 4.21 | 4.11 | 5.32 | 5.44 | 3.44 |
|  | MgO | 1.52 | 1.30 | 1.20 | 2.05 | 1.90 | 0.84 | 1.69 |
|  | CaO | 5.00 | 2.62 | 1.33 | 5.00 | 2.45 | 0.40 | 4.75 |
|  | Na_2_O | 1.28 | 2.14 | 1.98 | 1.86 | 1.59 | 0.33 | 2.16 |
|  | K_2_O | 1.98 | 2.65 | 2.70 | 2.27 | 2.46 | 2.50 | 2.29 |
|  | FeO | 1.40 | 1.10 | 0.90 | 1.25 | 0.80 | 0.80 | 1.40 |
|  | Si/Al | 7.88 | 8.05 | 8.97 | 9.36 | 7.59 | 6.07 | 10.31 |
|  | CIA | 67.64 | 58.20 | 60.44 | 57.83 | 64.64 | 82.48 | 53.48 |
|  | pH | 8.01 | 7.74 | 7.52 | 8.02 | 7.76 | 5.97 | 8.80 |
| **SOC content**  **(%)** | SOC | 1.10 | 1.35 | 1.07 | 0.62 | 0.79 | 0.97 | 1.00 |
| **SOC**  **composition**  **(R.A.)** | Alkyl | 0.31 | 0.27 | 0.23 | 0.34 | 0.29 | 0.28 | 0.34 |
|  | *N*-Alkyl | 0.12 | 0.11 | 0.10 | 0.14 | 0.11 | 0.10 | 0.11 |
|  | *O*-Alkyl | 0.23 | 0.22 | 0.21 | 0.21 | 0.18 | 0.19 | 0.18 |
|  | Di-*O*-Alkyl | 0.06 | 0.05 | 0.06 | 0.03 | 0.04 | 0.05 | 0.03 |
|  | Aromatic | 0.12 | 0.18 | 0.21 | 0.15 | 0.22 | 0.20 | 0.12 |
|  | O-Aromatic | 0.05 | 0.05 | 0.06 | 0.04 | 0.06 | 0.06 | 0.05 |
|  | Carboxylic | 0.11 | 0.12 | 0.13 | 0.09 | 0.11 | 0.12 | 0.16 |
|  | Alky/O-alkyl | 1.33 | 1.20 | 1.12 | 1.63 | 1.59 | 1.45 | 1.88 |
|  | 70-75/52-57 | 1.12 | 1.37 | 1.37 | 1.03 | 1.20 | 1.45 | 1.26 |
| **DOC content (mg C/g)** | DOC | 0.62 | 0.68 | 0.42 | 0.23 | 0.33 | 0.55 | 0.60 |
| **Absorbance** | SUVA_254_  (L mgC^-1^ m^-1^) | 1.58 | 1.80 | 1.93 | 1.86 | 1.60 | 0.84 | 2.01 |
|  | E2/E3 | 4.57 | 5.19 | 4.92 | 4.44 | 4.35 | 4.34 | 5.07 |
| **Fluorescence** | C1 (R.A.) | 0.35 | 0.42 | 0.41 | 0.40 | 0.38 | 0.35 | 0.45 |
|  | C2 (R.A.) | 0.36 | 0.32 | 0.33 | 0.24 | 0.24 | 0.11 | 0.22 |
|  | C3 (R.A.) | 0.29 | 0.25 | 0.26 | 0.35 | 0.38 | 0.54 | 0.33 |
|  | BIX | 0.59 | 0.53 | 0.58 | 0.52 | 0.57 | 0.68 | 0.55 |
|  | FI | 1.26 | 1.24 | 1.31 | 1.26 | 1.33 | 1.36 | 1.37 |
|  | HIX | 0.64 | 0.72 | 0.75 | 0.68 | 0.66 | 0.74 | 0.88 |

Table S1 continued.

|  |  | **GSS22** | **GSS23** | **GSS24** | **GSS25** | **GSS26** | **GSS27** | **GSS28** | **GSS29** |
| --- | --- | --- | --- | --- | --- | --- | --- | --- | --- |
| **Elemental**  **Content**  **(%)** | SiO_2_ | 68.23 | 59.80 | 69.11 | 60.93 | 66.15 | 58.87 | 61.04 | 63.16 |
|  | Al_2_O_3_ | 13.89 | 13.92 | 13.58 | 11.76 | 11.73 | 13.15 | 18.10 | 13.24 |
|  | Fe_2_O_3_ | 4.06 | 5.54 | 4.97 | 4.30 | 4.00 | 6.12 | 6.50 | 5.44 |
|  | MgO | 1.47 | 2.61 | 1.16 | 1.99 | 1.87 | 2.75 | 1.18 | 2.17 |
|  | CaO | 1.09 | 4.21 | 0.34 | 7.18 | 4.59 | 4.91 | 0.40 | 3.13 |
|  | Na_2_O | 2.84 | 1.91 | 0.83 | 1.74 | 1.90 | 1.22 | 0.29 | 1.32 |
|  | K_2_O | 2.97 | 2.64 | 2.48 | 2.28 | 2.18 | 2.37 | 2.83 | 2.31 |
|  | FeO | 0.60 | 1.50 | 0.80 | 1.30 | 1.20 | 1.70 | 1.20 | 1.61 |
|  | Si/Al | 8.34 | 7.29 | 8.64 | 8.79 | 9.57 | 7.60 | 5.72 | 8.10 |
|  | CIA | 58.46 | 60.36 | 74.42 | 58.94 | 57.67 | 66.65 | 81.84 | 65.93 |
|  | pH | 7.49 | 7.67 | 7.66 | 7.94 | 7.73 | 7.72 | 7.26 | 8.31 |
| **SOC content**  **(%)** | SOC | 0.30 | 0.50 | 0.50 | 0.58 | 0.73 | 0.90 | 1.15 | 1.12 |
| **SOC**  **composition**  **(R.A.)** | Alkyl | 0.33 | 0.36 | 0.26 | 0.30 | 0.26 | 0.27 | 0.28 | 0.27 |
|  | *N*-Alkyl | 0.13 | 0.13 | 0.11 | 0.11 | 0.11 | 0.11 | 0.10 | 0.11 |
|  | *O*-Alkyl | 0.23 | 0.20 | 0.21 | 0.21 | 0.23 | 0.19 | 0.23 | 0.26 |
|  | Di-*O*-Alkyl | 0.04 | 0.03 | 0.04 | 0.04 | 0.05 | 0.05 | 0.05 | 0.05 |
|  | Aromatic | 0.15 | 0.11 | 0.17 | 0.13 | 0.14 | 0.18 | 0.13 | 0.12 |
|  | O-Aromatic | 0.06 | 0.06 | 0.07 | 0.07 | 0.05 | 0.07 | 0.05 | 0.04 |
|  | Carboxylic | 0.06 | 0.11 | 0.14 | 0.14 | 0.15 | 0.13 | 0.15 | 0.15 |
|  | Alky/O-alkyl | 1.44 | 1.86 | 1.27 | 1.41 | 1.15 | 1.39 | 1.21 | 1.04 |
|  | 70-75/52-57 | 0.99 | 1.01 | 1.25 | 1.16 | 1.34 | 1.03 | 1.61 | 1.57 |
| **DOC content (mg C/g)** | DOC | 0.08 | 0.08 | 0.18 | 0.15 | 0.25 | 0.21 | 0.34 | 0.75 |
| **Absorbance** | SUVA_254_  (L mgC^-1^ m^-1^) | 1.28 | 1.37 | 0.94 | 2.37 | 2.22 | 1.99 | 1.05 | 1.29 |
|  | E2/E3 | 4.76 | 4.87 | 5.01 | 4.04 | 4.58 | 4.23 | 3.80 | 4.88 |
| **Fluorescence** | C1 (R.A.) | 0.42 | 0.37 | 0.32 | 0.39 | 0.41 | 0.39 | 0.34 | 0.38 |
|  | C2 (R.A.) | 0.33 | 0.41 | 0.29 | 0.21 | 0.27 | 0.33 | 0.06 | 0.14 |
|  | C3 (R.A.) | 0.25 | 0.22 | 0.39 | 0.41 | 0.32 | 0.28 | 0.60 | 0.48 |
|  | BIX | 0.67 | 0.65 | 0.71 | 0.60 | 0.52 | 0.57 | 0.63 | 0.58 |
|  | FI | 1.34 | 1.44 | 1.37 | 1.41 | 1.31 | 1.31 | 1.39 | 1.35 |
|  | HIX | 0.80 | 0.66 | 0.48 | 0.69 | 0.75 | 0.71 | 0.68 | 0.76 |

* Abbreviations:

CIA, chemical index of alteration; 70-75/52-57, area ratio of 70-75/52-57 ppm; SUVA_254_, the specific ultraviolet absorbance at 254 nm; E2/E3, absorbance at 254 nm divided by the absorbance at 365 nm; C1 - C3, relative abundance of fluorescence components 1 to 3; BIX, biological index; FI, fluorescence index; HIX, humification index; R.A., relative abundance.

Table S2: Regional signatures of 2087 formulae occurring in 90% of soil samples.

|  | **Region I** | **Region II** | **Region III** | **Region IV** |
| --- | --- | --- | --- | --- |
| Formulae | C_16.71_H_17.13_O_8.12_  N_0.77_S_0.00_ | C_19.29_H_25.73_O_6.18_  N_0.34_S_0.00_ | C_14.48_H_22.04_O_9.33_  N_1.01_S_0.01_ | C_16.19_H_27.18_O_6.66_  N_0.80_S_0.03_ |
| Average *m*/*z* | 358.56 | 360.96 | 359.45 | 340.44 |
| Average O/C | 0.49 | 0.32 | 0.65 | 0.42 |
| Average H/C | 1.02 | 1.33 | 1.53 | 1.68 |
| Average N/C | 0.05 | 0.02 | 0.07 | 0.06 |
| Average S/C | <0.01 | <0.01 | <0.01 | <0.01 |
| Average AI_mod_ | 0.39 | 0.27 | 0.02 | 0.02 |
| Average DBE | 9.53 | 7.59 | 4.96 | 4.00 |
| Average NOSC | 0.12 | -0.62 | -0.01 | -0.68 |
| **Biochemical composition** | | | | |
| % Lipid | 5.5 | 54.2 | 28.6 | 74.9 |
| % Protein | 10.7 | 5.3 | 32.4 | 25.1 |
| % Amino sugar | <0.1 | <0.1 | 26.9 | <0.1 |
| % Carbohydrate | <0.1 | <0.1 | 5.5 | <0.1 |
| % Phytochemical | 83.8 | 40.5 | 6.6 | <0.1 |

* Abbreviations: AI_mod_, modified aromaticity index; DBE, double-bond equivalent; NOSC, nominal oxidation state of carbon. Phytochemical compounds here refer to oxy-aromatic compounds. Region definitions: Region I (H/O ≤3 and AI_mod_ ≥0.1), Region II (H/O >3 and AI_mod_ ≥0.1), Region III (H/O ≤3 and AI_mod_ <0.1) and Region IV (H/O >3 and AI_mod_ <0.1).


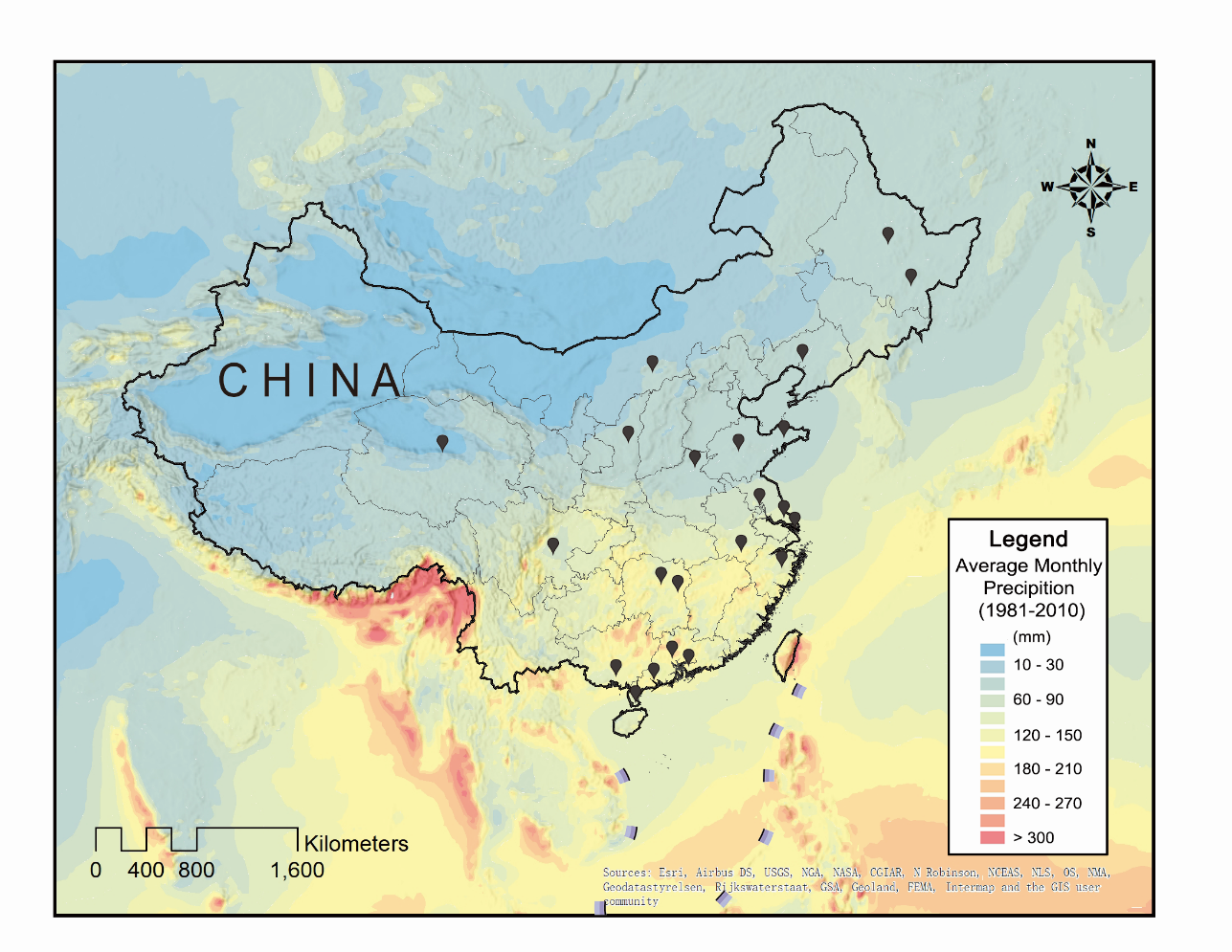


Figure S1: Map showing the locations of the 22 Chinese reference soils. However the exact locations of each standard reference soils were not available, therefore symbols on the map just show the general region. Color scale indicates the average monthly precipitation from 1981 to 2010.





Figure S2: Relationships of the dissolved organic carbon yield with soil organic carbon content, ^13^C-NMR compositions, soil elemental composition and soil pH. 70-75/52-57, area ratio of 70-75/52-57 ppm; CIA, chemical index of alteration.


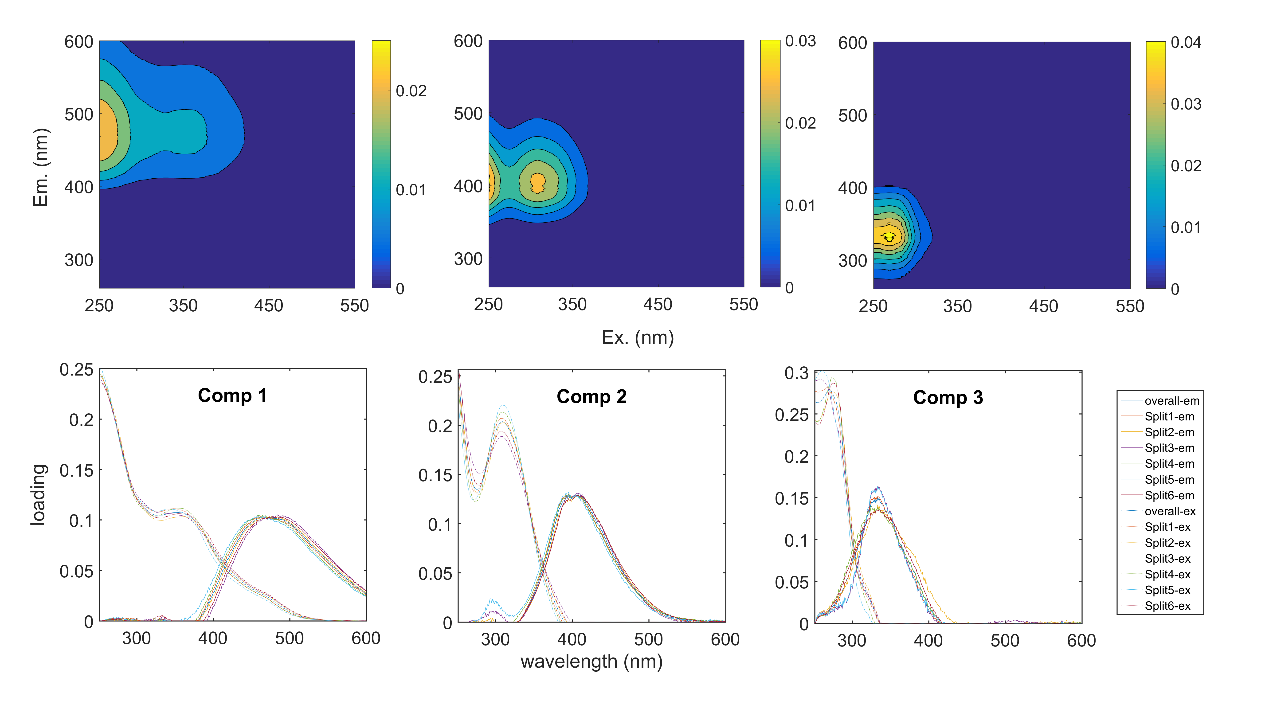


Figure S3: Fluorescence signatures of three PARAFAC components. Line plots show split-half validations for each component.





Figure S4: Correlations for the relative abundances of the common DOM formulae versus all soil DOM formulae in four regions. Red line indicates the 1:1 line; black line is linear regression line. Region definitions: Region I (H/O ≤3 and AImod ≥0.1), Region II (H/O >3 and AImod ≥0.1), Region III (H/O ≤3 and AImod <0.1) and Region IV (H/O >3 and AImod <0.1).





Figure S5: Molecular associations between individual molecules and soil elemental composition. DOM formulae having significant Spearman correlations (*P*<0.1) with the elemental composition are plotted. Red color indicates a positive correlation, and blue color indicates a negative correlation. CIA, chemical index of alteration.





Figure S6: Molecular associations between individual molecules and soil DOM properties. DOM formulae having significant Spearman correlations (P < 0.1) with the DOM properties are plotted. Red color indicates a positive correlation, and blue color indicates a negative correlation. %C1 - %C3, relative abundance of fluorescence components 1 to 3; SUVA_254_, the specific ultraviolet absorbance at 254 nm; BIX, biological index; HIX, humification index; FI, fluorescence index; DOC/TDN, ratio of DOC to total dissolved nitrogen.





Figure S7: Molecular associations between individual molecules and soil organic matter properties. DOM formulae having significant Spearman correlations (*P*<0.1) with soil organic matter properties are plotted. Red color indicates a positive correlation, and blue color indicates a negative correlation. 70-75/52-57, area ratio of 70-75/52-57 ppm;





Figure S8: Relationships between the relative abundance of labile molecules above the molecular lability boundary (% MLB_L_) with soil organic carbon content, ^13^C-NMR compositions, soil elemental composition and soil pH. 70-75/52-57, area ratio of 70-75/52-57 ppm; CIA, chemical index of alteration.





Figure S9: Relationships between the modified aromatic index (AImod) with soil organic carbon content, ^13^C-NMR compositions, soil elemental composition and the pH. 70-75/52-57, area ratio of 70-75/52-57 ppm; CIA, chemical index of alteration.





Figure S10: Redundancy analysis showing the relationship between soil elemental composition, SOM composition and soil DOM optical characters. Circular points indicate specific soil samples.





Figure S11: Principle component analyses of soil elemental composition, soil organic matter components and soil DOM characters. The lower panels display the related sample scores.
